# Hydrogel Design to Understand and Guide 3D Cell Migration

**DOI:** 10.1007/s40883-025-00395-z

**Published:** 2025-04-24

**Authors:** Karen L. Xu, Robert L. Mauck, Jason A. Burdick

**Affiliations:** 1 Department of Bioengineering, University of Pennsylvania, Philadelphia, PA 19104 USA; 2 Center for Engineering Mechanobiology, University of Pennsylvania, Philadelphia, PA 19104 USA; 3 Department of Orthopaedic Surgery, Perelman School of Medicine, McKay Orthopaedic Research Laboratory, University of Pennsylvania, Philadelphia, PA 19104 USA; 4 Translational Musculoskeletal Research Center, Corporal Michael J. Crescenz VA Medical Center, Philadelphia, PA 19104 USA; 5 BioFrontiers Institute, University of Colorado Boulder, Boulder, CO 80309 USA; 6 Department of Chemical and Biological Engineering, University of Colorado Boulder, Boulder, CO 80309 USA

**Keywords:** Cell migration, Microinterfaces, Cell-material interfaces, Engineered matrices

## Abstract

**Purpose:**

The extracellular environment is critical for cell migration in three-dimensions (3D), which has been understudied when compared to cell migration on two-dimensional (2D) substrates. In 3D, cells must degrade or remodel their surroundings to overcome barriers to migration or find paths that act as migration routes.

**Methods:**

We performed a literature search for studies related to the engineering of hydrogels to understand and control cell migration.

**Results:**

This review highlights the cell-intrinsic machinery that is required for migration, describes how cell migration can be modeled in vitro, and provides examples where hydrogels have been designed with permissive extracellular cues that enhance cell migration for biomedical applications.

**Conclusions:**

Hydrogels can be engineered to mimic many features of the extracellular space to help us better understand the interplay between cells and their environment and interpret how these complex processes support or limit cell migration. With this understanding, hydrogels can be designed to guide cellular migration, particularly in the context of tissue repair and regenerative medicine.

**Lay Summary:**

Cell movement is important in both healthy and diseased tissues. An understanding of how cells migrate and the development of methods to control their migration can be utilized to improve patient therapies in the future in applications such as tissue repair and regeneration. Hydrogels are water-swollen materials that mimic many features of tissues. This allows their use to understand how cells respond to various features in their environment, as well as for therapeutic materials in tissue repair. This review highlights advances on these topics.

## Introduction

Cell migration is required for many biological processes found during development, disease, wound healing, and regeneration [1]. For example, in cancer, cell metastasis results in disease spreading and reduces the efficacy of treatment, whereas fibroblast migration and matrix deposition during wound healing after injury help to stabilize the tissue. The ability of a cell to migrate depends on the reciprocity between intracellular states and extracellular environments, the interplay of which determines the efficiency with which cells traverse through their environment. Migration has been well characterized for cells on two-dimensional (2D) substrates and although we can learn much about migration based on these 2D studies, there are differences when cells migrate in three dimensions (3D), such as the introduction of confining barriers that cells must overcome to migrate forward in 3D [2]. As such, cells may be primed (via signaling pathways or extracellular soluble factors) to migrate, but still be unable to migrate due to physical impediments. In vivo, cellular paths for migration exist naturally due to the tissue structure or are created through remodeling of the extracellular environment, either through cell-mediated physical path formation or protease-mediated degradation. An appreciation of the permissiveness of extracellular environments to cell migration may be critical to better understand cell migration in undesirable (cancer metastasis) and desirable (regenerative medicine) scenarios.

Numerous assays have been developed to support and quantify cell migration in 3D. Primarily, the transwell or Boyden chamber invasion assays consist of two separate compartments separated by a porous membrane. Soluble factors [3] or other cells [4] may be placed in the bottom compartment of the transwell insert, with a migratory cell type seeded atop the insert. Cells that traverse to the opposite side of the insert can be quantified to investigate the effect of paracrine factors on cell migration. Additionally, inset pore size can be tuned to explore how confinement affects cell migration [5, 6]. Despite their utility in biology, the rigid substrates and porosity presented via a single layer that are used in these assays may not mimic the biochemical and biophysical features that are observed within cellular extracellular microenvironments.

To address this, engineered hydrogels present opportunities to both understand the complex extracellular environments that support migration in vivo and to serve as 3D platforms to control migration for specific biomedical applications. Hydrogels are water-swollen crosslinked polymer networks that can be tuned to introduce biochemical (cell-adhesion moieties and protease-degradable sites) or biophysical (stiffness, stress relaxation, topologic cues) features to cells [7]. These features can be decoupled from each other and systematically altered to explore how targeted signals within cellular environments influence cell migration. Hydrogel mesh sizes are dependent on the type of hydrogel and the extent of crosslinking and are typically below 20 nm [8]. While this permits solute transport and supports 2D migration along hydrogel surfaces, it is orders of magnitude below the pore sizes needed for cell migration. Thus, to support migration in 3D, hydrogels must be engineered with either cell-sized paths for migration or with features (e.g., degradation) that respond to cellular behaviors.

Here, the current understanding of the cell machinery that is required to prompt cell migration and the various signals that induce directional migration are outlined. Next, the use of hydrogels to explore 3D migration are highlighted, including techniques such as patterning and microfluidics. Finally, hydrogel-based strategies that implement extracellular cues that permit cell migration are explored as translational materials for tissue repair. Although not comprehensive of all studies performed on this topic, this review is meant to highlight important examples that allow the reader to understand the breadth of work on hydrogel engineering for 3D migration.

## Fundamentals of Cell Migration in 3D

To migrate, cells must interact (via traction or propulsion) with their extracellular environments. In single cells, mesenchymal migration (fibroblasts, stem cells) relies heavily on integrin-based adhesions to couple the extracellular environment with actin cytoskeletal connections via the strengthening of focal adhesions [9]. These connections then act as complex signaling hubs that induce signaling pathways to support migration. In contrast, amoeboid migration (neutrophils, T-cells) relies on limited cell adhesion with their extracellular environments, with any adhesion consisting of largely integrin-independent mechanisms. In these cases, actin polymerization (as well as hydrostatic pressure) supports membrane protrusion, generating forces to enable migration [10]. While not required for migration in these cell types, actomyosin contractility is increasingly utilized within confining environments [11]. Other strategies that cells may use to migrate include the use of a “nuclear piston,” in which the nucleus compartmentalizes pressure to enable cell migration [12]. While these modes of migration have been discretely categorized, cells often utilize multiple strategies concurrently or alter the mode of migration based on the perceived biochemical and physical milieu. Most of our fundamental understanding on migration has been performed in 2D, representing how cells move along a planar surface, which has been reviewed previously [13, 14]. Although many of these mechanisms are applicable to 3D migration, the addition of confinement adds additional complexity that must be considered.

Cells are known to exhibit directional migration in 3D based on gradients of signals in their environment [15]. The most well studied cue is chemotaxis, in which soluble factors are differentially presented to induce directional migration. Prompting factors can include chemokines, growth factors, morphogens, or even extracellular vesicles [16, 17]. As discussed further below, a variety of fabrication methods can be used to introduce gradients into hydrogels such as through degradation and release of embedded factors [18]. This strategy not only enables the exploration of chemotactic efficacy [19, 20] but also supports the incorporation of chemotactic cues into hydrogels to promote endogenous cell migration [21].

Other gradient-based cues have been explored extensively in 2D, often with cells on glass or engineered substrates [22]. For example, haptotaxis occurs in response to biochemical cues bound to the extracellular environment, such as adhesive sites or peptide sequences on extracellular matrix (ECM) molecules [23, 24]. The study of haptotaxis in 3D has been largely limited to theoretical results [25], likely due to the complex fabrication methods required to introduce bound gradients to cells in vitro. Similarly, durotaxis is the response of a cell to variations in stiffness, which is also challenging due to limited fabrication methods. In one example, a 3D stiffness gradient was introduced through compressing wedge-shaped collagen and demonstrated that cells migrate towards higher stiffnesses in 3D, similar to their 2D response [26]. Yet, cells placed in matrices of increased stiffness in 3D often migrate more slowly [27–29], which highlights the challenge of decoupling signals such as stiffness and matrix density in 3D systems.

Galvanotaxis, or directional migration in response to electric potentials, has also largely been limited to 2D studies [30–32] due to the technical difficulties that arise in 3D such as cytotoxic temperature rises with chamber depth or drifting of 3D constructs [33]. Interestingly, brain tumor initiating cells migrated in opposite directions in response to electric potential based on dimensionality (2D supported migration towards the anode and 3D supported migration towards the cathode), and myosin II facilitated galvanotaxis in 3D, but not in 2D [34]. Clearly, cues that prompt directional migration in 2D (e.g., chemotaxis, haptotaxis, durotaxis, galvanotaxis) must be further explored in 3D. Directional migration has been reviewed extensively elsewhere [15].

## Hydrogels to Model Features that Support 3D Cell Migration

**Fig. 1:**
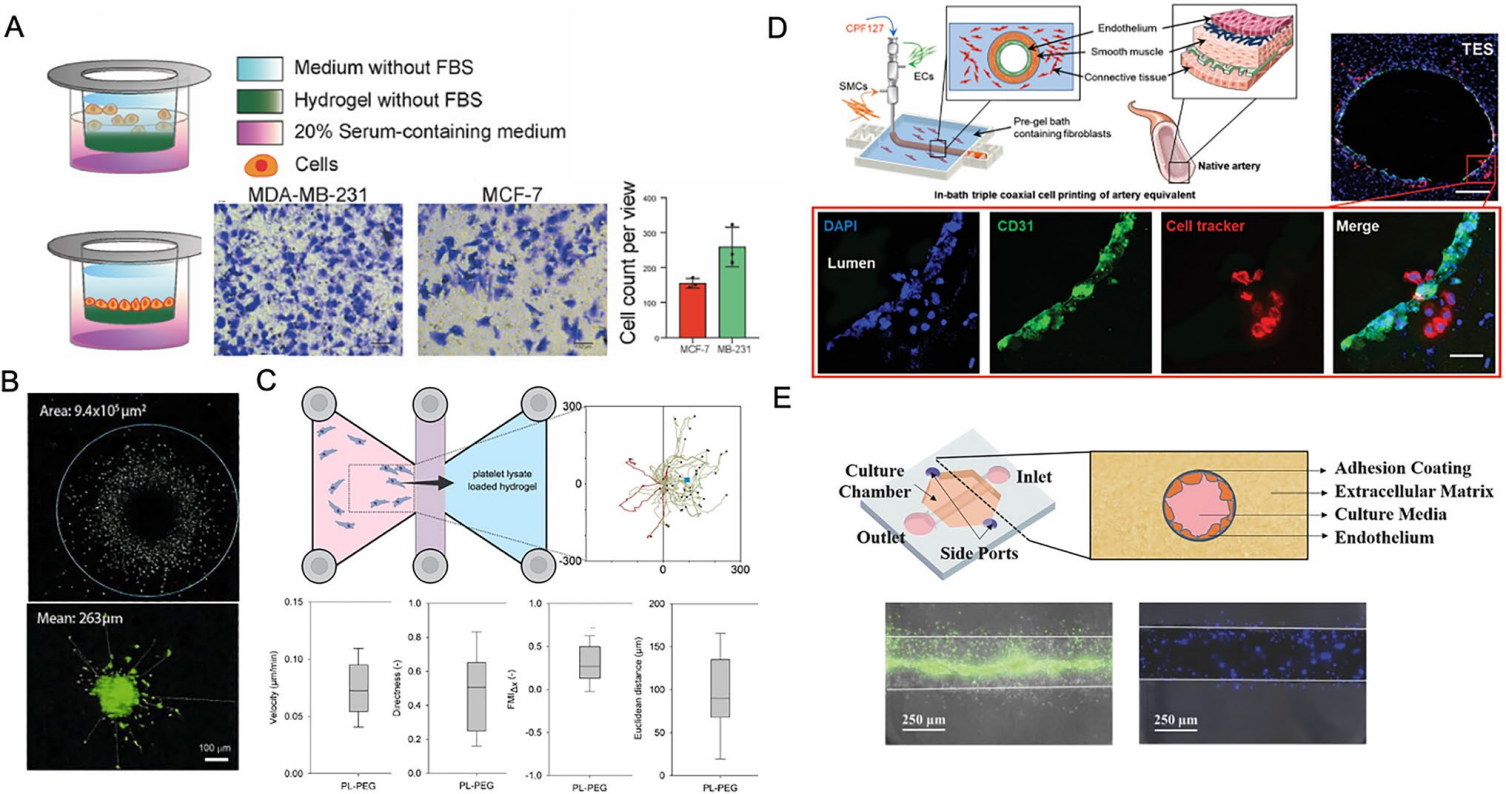
Examples of cell migration through hydrogels in vitro. **A** Transwell inserts facilitate the assessment of cell migration through hydrogels, enabling differential migration of various cell types, including MDA-MB-231 and MCF-7 cells. Adapted with permission from [35]. **B** Representative example of spheroid outgrowth into a hydrogel, allowing quantification of outgrowth from spheroid body. Adapted with permission from [40]. **C** Live-cell tracking to image cell migratory paths towards a hydrogel releasing platelet lysate as a chemotactic signal, including quantification of migration velocity, directness (displacement/total path length), forward migration index (FMI_Δ*x*_), and Euclidean distance. Adapted with permission from [41]. **D** 3D bioprinting to mimic complex ECM features, in which coaxial printing to pattern cells is used to explore conditions that support monocyte (cell tracker) infiltration through endothelial cell layers (CD31, DAPI). Scale bar = 200 μm, zoom-out, 20 μm inset. Adapted with permission from [42]. **E** Microfluidics enable the engineering of complex cellular environments, including to model neutrophil migration across endothelial cell layers into ECM (green, calcein AM; blue, nucleus). Adapted with permission from [43]

Hydrogels are advancing as 3D platforms to investigate cell migration. In the simplest form, hydrogels can be introduced into the aforementioned transwell platforms, specifically to explore migration through hydrogels [35, 36]. Numerous examples exist, such as to investigate DNA hydrogels that differentially support cancer cell migration (Fig. 1A) [35], the addition of carbon nanotubes to a glycol chitosan hydrogel for fibroblast migration during wound healing [37], the introduction of a transglutaminase crosslinked gelatin to increase the retention of injected stem cells post-MI [38], or the inclusion of platelet rich-plasma (PRP) to a gelatin methacryloyl hydrogel to support osteochondral regeneration through M2 macrophage polarization [39].

Cell migration through 3D environments can also be directly investigated through the embedding of cell pellets or spheroids in hydrogels [40]. Cell clusters serve as point sources of cell outgrowth, in which radial migration distances can be quantified and compared to the original spheroid size. These studies have explored the screening of pharmacologic agents for neuroregeneration (Fig. 1B) [40], chemotherapeutic agents for cancer metastasis [44], chemotactic agents for wound healing [45], and permissive cellular environments for angiogenesis [46]. Similar to spheroid invasion studies, cells [47] or explants [48] can be placed on one side of the hydrogel or between two hydrogel layers, and cell invasion quantified with imaging [49].

With the greater accessibility of time-lapse technologies, cells can now be placed within or in proximity to 3D hydrogels and tracked over time, enabling the user to explore parameters such as migration speed and persistence towards chemotactic signals (e.g., platelet lysate) (Fig. 1C) [41, 50–52]. Innovative platforms build on simple time-lapse imaging of random cell movement to now provide clearer outcomes of cell migration. For example, cells embedded in hydrogels can be placed atop micropillars, in which cells that migrated through the hydrogel to the micropillar surface can be quantified [53]. Magnetic levitation may also be used to create 3D ring-shaped cultures, where ring closure can be assessed over time as a proxy of cell migration to assess toxicity to various molecules (e.g., decreased ring closure suggests increased toxicity) [54].

Higher-order fabrication technologies may also be used with hydrogels to explore cell migration in 3D. For example, photografting and photodegradation are used to soften regions of hydrogels that better support alignment of migrating cells [55] or directionally instruct organoid growth [56]. Bioprinting may be used to introduce more complex environments into hydrogels to better mimic native 3D structures. For example, leukocyte-mediated cell migration from vessels can be modeled with the spatial deposition of bioinks that match features of the ECM or cellularity of tissues (Fig. 1D) [42]. These models can be used to explore migration through various tissue layers towards bioprinted chemotactic cues [57]. 3D printing may also be used to mimic the gradual variation in bone morphology, in which gradients in scaffold concentration and composition support MSC infiltration and osteogenic differentiation [58]. Additionally, the precise deposition of various bioinks can enable co-culture studies to investigate the impact of cell–cell distance on angiogenesis (endothelial cell migration), in which a distance less than 200 μm between endothelial cells and mesenchymal stromal cells supports increased angiogenesis and regeneration of bone defects when compared to co-cultures separated by larger distances [59].

Microfluidics may also be used to introduce 3D environments to explore cell migration. Most commonly, sophisticated designs can mimic the complex and potentially competing chemotactic cues that induce cell migration [60]. For example, microfluidics containing hydrogels can be used to explore neutrophil extravasation from iPSC-derived endothelial microvessels (Fig. 1E) [43]. Microfluidics can also be used to apply direct current electric field gradients [61] or to facilitate downstream imaging and transcriptomic analysis of invaded cells [62]. Like 3D printing, microfluidics may also be used to support co-culture studies, in which microfluidic reservoirs may separate various cell types and their subsequent interactions then monitored [63]. Higher order complexities can be recapitulated with microfluidics in organ-on-a-chip studies, in which cell types and soluble factors are spatially distinct to mimic native tissues. These devices can be used to screen pharmacologic agents or to explore in vitro how complex cell-ECM interactions influence disease progression [64]. Several comprehensive reviews on assays of 3D migration have been previously published [65, 66].

## Engineering Hydrogels that are Permissive to Cell Migration

**Fig. 2:**
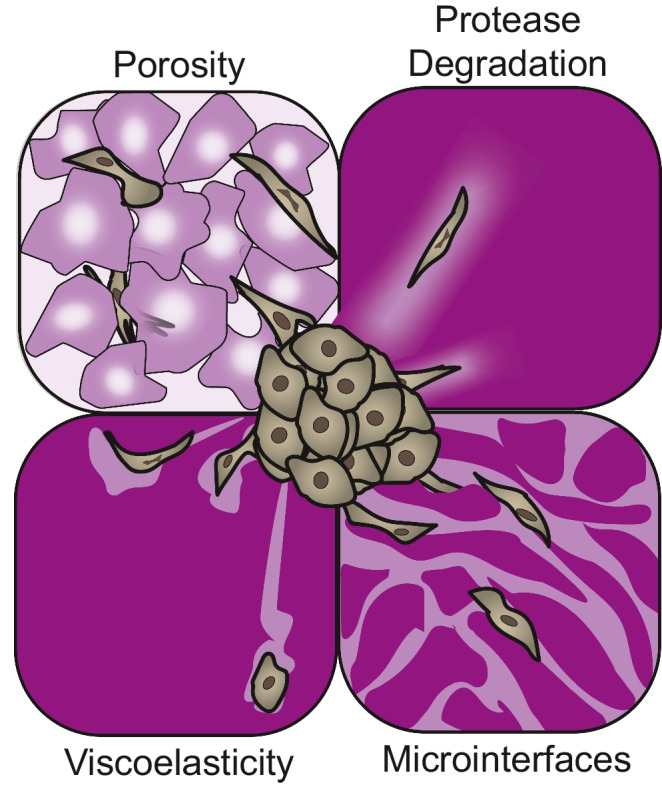
Schematic highlighting hydrogel cues that permit cell migration. Hydrogels can be engineered to support cell-mediated (e.g., protease degradation, viscoelasticity) migration through biochemical or biophysical cues or fabricated with pre-determined (e.g., porosity, microinterfaces) paths to guide cell migration

Migration will not occur unless the extracellular environment is permissive to cell movement; thus, hydrogels have been engineered to enable such behaviors. Here, we explore four major strategies to date that have been used to support 3D migration in hydrogels, including (i) proteolytic degradation, (ii) viscoelasticity/plasticity, (iii) porosity, and (iv) microinterfaces (Fig. 2). We focus on single cell migration that relies on adhesion-based mesenchymal modes of migration, although significant work has also been conducted to explore alternate adhesion-independent amoeboid modes of migration [67].

### Protease Degradation to Support Migration in 3D

The ECM consists of a large range of diverse constituents, including collagens, proteoglycans, elastins, and glycoproteins [68]. ECM surrounding cells is constantly being remodeled, including through matrix metalloproteinases (MMPs), disintegrin and metalloproteinases (ADAMs), or disintegrin and metalloproteinase with thrombospondin motifs (ADAMTS), each with affinity to specific ECM proteins [69]. Cell migration, when confronted with obstructing ECM, may rely on remodeling of the ECM via protease activity. For example, in biological processes such as angiogenesis and metastasis, degradation of the endothelial basement membrane is required to support lumen formation or cell invasion, respectively [69, 71, 70]. In addition to removing obstacles, degradation supports the introduction of “highways” that can result in even greater cell migration, including with the orientation of collagen fibers to provide directional migration cues [69]. Here, we highlight several strategies to incorporate protease-mediated degradation into hydrogels.

**Fig. 3:**
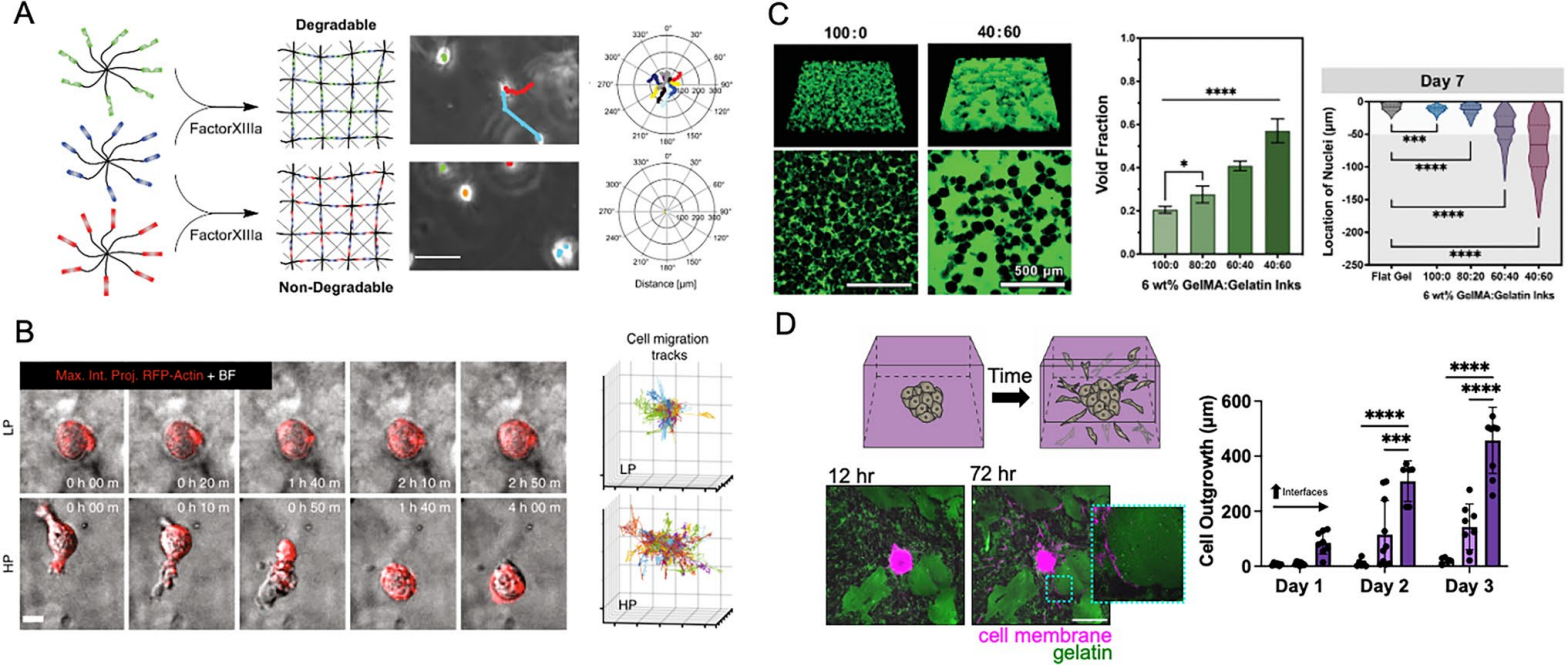
Engineering permissive extracellular cues into hydrogels to support cell migration. **A** Poly(ethylene glycol) hydrogels (left panel) with engineered degradation support increased cell migration (middle panel, representative views; right panel, visualization of cell trajectories). Scale bar = 50 $\upmu$ m. Adapted with permission from [76]. **B** Cells in HP (high plasticity) hydrogels (compared to LP, low plasticity) remodel their extracellular environments towards increased cell migration. Scale bar = 10 μm. Adapted with permission from [84]. **C** Hydrogels with increased porosity (green) exhibit increased cell densities within hydrogels compared to those with lower porosity, where cells are concentrated near the hydrogel surface. *p < 0.05, ***p < 0.001, ****p < 0.0001. Adapted with permission from [85]. **D** Spheroids embedded within hydrogels with extensive microinterfaces (compared to homogenous hydrogels) exhibit increased cell migration. Scale bar = 200 μm. ***p < 0.001, ****p < 0.0001. Adapted with permission from [86]

Given that natural hydrogels (e.g., gelatin [72], Matrigel [73]) have native cell-adhesive and inherent protease degradation sites, they provide inspiration for the formation of synthetic hydrogels with such features. Specifically, the advantages of synthetic hydrogels, such as increased control over hydrogel properties and the ability to decouple properties such as adhesivity and stiffness, can be combined with those of natural hydrogels by introducing peptide crosslinkers that are sensitive to enzymes. For example, peptides degradable by elastase, collagenase, and plasmin have been introduced to hydrogels formed from polymers such as inert poly(ethylene glycol) (Fig. 3A) [74–76] or dextran [77].

These synthetic materials can be combined with other assays to probe changes in local properties with degradation, such as with pericellular microrheology to define local mechanics [78] or with traction force microscopy to measure cell-mediated forces during degradation [79]. Degradation sites can also be engineered into electrospun fibers to introduce ECM-mimetic fibrillar structures into hydrogels [80]. Other forms of degradation (e.g., hydrolysis) are often insufficient to support cell migration since they occur throughout the material and not locally [81]. Degradation can also be harnessed to deliver drugs or other soluble factors, either when covalently conjugated onto the hydrogels or when embedded within hydrogels. For example, hydrogels can be designed to release chemotactic factors to encourage cell migration, such as from dense connective tissues that would otherwise not support migration [18]. In addition to soluble factor release, polymer byproducts of hydrogel degradation may be directly harnessed for cell migration, such as with digested decellularized extracellular matrices [82]. The cytocompatibility of degradation byproducts must also be considered, as chemicals may be inert when immobilized, but toxic once released [83]. Such materials with toxic byproducts should generally be avoided in biomedical studies.

### Viscoelasticity and Viscoplasticity in Migration

Tissues are often viscoelastic, with time-dependent mechanical properties. In response to mechanical stimulus, viscoelastic tissues will elastically deform and exhibit time-dependent creep. Additionally, viscoplastic materials exhibit permanent changes when they exceed a material yield stress [2]. When mechanical stimuli are removed, tissues may undergo stress relaxation, in which stored energy dissipates over time. The ECM plays a large role in dictating these mechanical responses, as the complex interplay between collagen crosslinks, entanglements, and elastin fibers as well as fluid flow through permeable tissue (poroelasticity) may contribute to energy dissipation [87]. Importantly, changes in viscoelasticity and viscoplasticity often accompany pathologic states, highlighting the need to understand their influence on cell behaviors like migration [88].

Viscoelasticity and viscoplasticity have been introduced into hydrogels to understand their influence on cells. For 2D studies, polyacrylamide hydrogels have been engineered to maintain their elastic modulus while varying their loss modulus by tuning the ratio of bis-acrylamide and acrylamide [89] or by incorporating linear polyacrylamide chains [90], whereas alginate hydrogels can be formed with both covalent crosslinking and ionic crosslinking to introduce elastic and viscoelastic properties, respectively [91]. These systems have shown that key mechanotransduction pathways are activated in cells based on energy dissipation of the surrounding substrate and numerous theoretical models, including the motor-clutch model, have been used to explore the balance between hydrogel relaxation and focal adhesion binding [92]. In 3D, confinement activates machinery that is distinct from that required in 2D migration. 3D interpenetrating networks (IPNs) (in which multiple networks are crosslinked throughout each other) can be engineered to decouple viscous and elastic contributions of the extracellular environment to modulate cell behavior, such as increased cell spreading with increased viscoelasticity. Examples include varying concentrations of aldehyde or benzaldehyde modified hyaluronic acid [93], alginate modified with varying molecular weights of PEG hydrogels [94], a synthetic system based on supramolecular interactions between cyclodextrin and adamantane or cholic-acid [95], and a PEG-based hydrogel with dynamic covalent boronate bonds formed from interactions between boronic acids and cis-1,2-diols [96].

While similar, cell migration relies on cell signaling mechanisms distinct from cell spreading. Few studies have specifically explored the role of viscoelasticity and viscoplasticity on cell migration in 3D, despite the physiologic relevance of these properties. In one study, viscoplasticity, or the permanent deformation after viscoelastic response to perturbations was engineered with an alginate and basement membrane IPN (Fig. 3B) [84]. Cells in these environments mechanically remodel their pericellular environment when confronted with a confining, but permissive 3D environment, particularly with invadopodia protrusions. Other systems can be engineered to confirm these trends such as a low-molecular weight alginate (fast-relaxing) that supports increased cell outgrowth from mesenchymal stromal cell spheroids when compared to high-molecular weight alginate (slow-relaxing) [97]. Additionally, increased epithelial-to-mesenchymal cell transitions are observed in hydrogels with high stress relaxation [98].

Other viscoelastic hydrogels have also been developed. For example, natural hydrogels that demonstrate viscoelastic behaviors include collagen, Matrigel, and fibrin [99]. Synthetic strategies to introduce viscoelasticity include dynamic covalent imine and acylhydrazone bonds with gelatin and dextran backbones [100], photoinitiated thiol-ene reactions that tune excess thiol concentration and thereby stress relaxation [101], and dynamic alkyl-hydrazone vs. benzyl-hydrazone bonds [102]. These systems have not been used to specifically explore cell spreading or migration but may be valuable tools for future studies. It should be noted that directional migration in viscoelastic and viscoplastic hydrogels will require a directional signal, such as gradients in mechanical properties or the release of a chemotactic signal.

### Porosity to Define Migratory Paths in 3D

The extent of porosity found in vivo is dependent on the tissue type. For example, fibrillar collagen in mice mammary glands can have pores greater than 20 μm, periosteum has pores less than 10 μm, and dermis can range from 1 to 20 μm, with ease of cell migration through tumors based on these gap sizes [103]. This variability in pore sizes underscores the importance in exploring the role of porosity to support cell migration. The ability of a cell to traverse through pore spaces in the ECM is regulated by the nucleus (the stiffest organelle of the cell) and its deformation capacity [104, 105]. Strategies have been explored to increase cell migration by reducing the stiffness of the nucleus, both by reducing the nuclear protein Lamin and by decreasing the density of heterochromatin content [106]. However, alterations in cell machinery may have off-target effects on genetic expression, so increasing porosity in engineered hydrogels may be a viable approach to enhance migration.

**Fig. 4:**
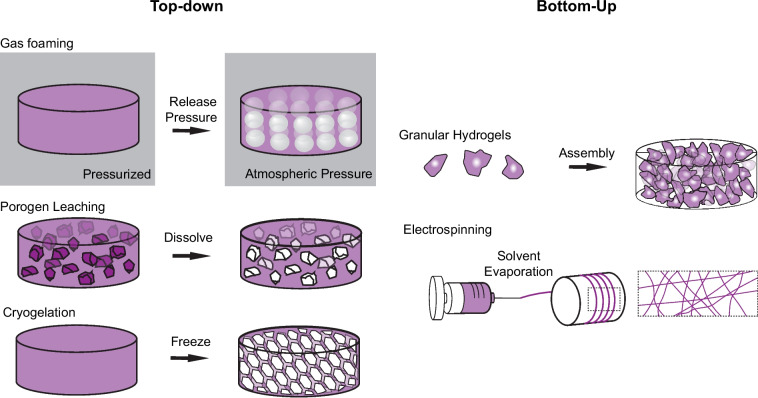
Fabrication approaches to introduce porosity into synthetic environments. Porosity can be fabricated into hydrogels via top-down approaches or bottom-up approaches. Top-down approaches include gas foaming, in which hydrogels are pressurized and gas nucleation forms pores upon depressurization; porogen leaching, in which sacrificial components are removed to introduce pores; and cryogelation, in which water crystallization with freezing introduces pores. Bottom-up approaches include the assembly of microgels (granular hydrogels) or electrospinning

Given its well characterized importance in supporting cell migration, many strategies have been used to fabricate microporous hydrogels [107]. These pore structures are often much larger than those found within tissues, but they eliminate potentially restrictive confinement barriers to allow cells to migrate through a 3D material. These methods primarily rely on top-down manufacturing, in which porosity is introduced into otherwise bulk materials (Fig. 4). Examples include particle leaching where sacrificial particles, such as sugar or gelatin, are embedded during initial hydrogel formation and then later removed. Water crystallization can be used to introduce pores into hydrogels to form cryogels with freezing, including with the introduction of anisotropic pores [108]. Finally, gas foaming processes introduce pores via the expansion of gas nuclei when hydrogels are transferred from high pressure environments to atmospheric pressure [109].

Bottom-up approaches are also used to introduce porosity into hydrogels. As one example, electrospinning is used to fabricate hydrogel fibers to mimic the native fibrous topology of ECM. Electrospinning concentrates the polymers along dense fibers, inherently introducing pores [110]. Cells can either migrate along the surface of this porous structure (termed 2.5D migration), particularly when the pore sizes are too small, or through the fibrous scaffold, which would be considered 3D migration. Electrospun fiber porosity can be tuned through altering fiber alignment, where fibers with random orientation have increased porosity and cell integration when compared to aligned fibers [111]. Additionally, alterations in the fiber diameter also influences porosity and cell migration [112]. To further enhance porosity within dense electrospun networks, porosity can also be introduced through sacrificial fibers that are removed with further processing (similar to particle leaching) [113]. To support cell infiltration into the fiber networks themselves, hydrolytically degradable fibers may be tuned to release PDGF, a chemotactic agent, thereby creating a gradient of this agent from the defect edge to the center [18]. Similarly, electrospinning can be combined with other previously mentioned strategies to increase porosity, such as the introduction of salt leaching [114]. For more information about other advanced fabrication methods to introduce enhanced electrospun fiber porosity, please refer to [115, 116].

Another modular approach to introduce pores into hydrogels is the assembly of hydrogel microparticles (microgels) into granular hydrogels [117]. This packing of microgels introduces extensive interconnected porosity into 3D systems and is tunable based on the shape and stiffness of the assembled particles [46, 118–120], as well as the method of particle packing [121]. Porosity can be further engineered through the introduction of sacrificial polymers, such as gelatin, in which incubation at 37ºC supports void formation through gelatin dissociation (Fig. 3C) [85]. Additionally, various particles can be combined, thereby increasing the modularity and complexity in these hydrogels. This strategy has enabled microgel assemblies to release different molecules where the kinetics and extent of drug release are based on the ratio of different microgels [122]. Clearly, the tunable nature of granular hydrogels increases the complexities that can be introduced into this system.

In addition to these methods, the assembly of natural hydrogels (including collagen and fibrin) into fibrillar structures results in porosity. For example, cell migration decreases with increasing collagen concentration due to decreased porosity [123]. Collagen networks have also been used to characterize cell migration in anisotropic structures in 3D [124], to explore the role of strain stiffening in the densification and alignment of fibers to guide migration [125], and to highlight matrix density as a determinant of migration types in lymphocytes [126], among other studies [127–130]. Fibrin may also be used to study 3D migration [131, 132], including confirming that decreased migration occurs with increasing matrix density and that the delivery of chemotactic agents such as CTGF or TGF-beta from poly(lactic-co-glycolic acid) (PLGA) increases migration [133]. It should be noted that these responses are likely quite complex, where matrix density not only influences porosity of a natural material, but features such as degradability and matrix crosslinking. For example, multiple signals often combine to influence migration, such as the combination of porosity and MMP activity to accelerate cell migration [123, 134] and the investigation of viscoelastic fibrils that deform on different time-scales influenced by interstitial fluid within the fibril network [135]. In one study, this was decoupled by altering the stiffness of individual synthetic fibers while maintaining porosity and fiber dimensions, implicating fiber stiffness as one important feature in 3D migration [48].

### Microinterfaces as Pathways in 3D

**Fig. 5:**
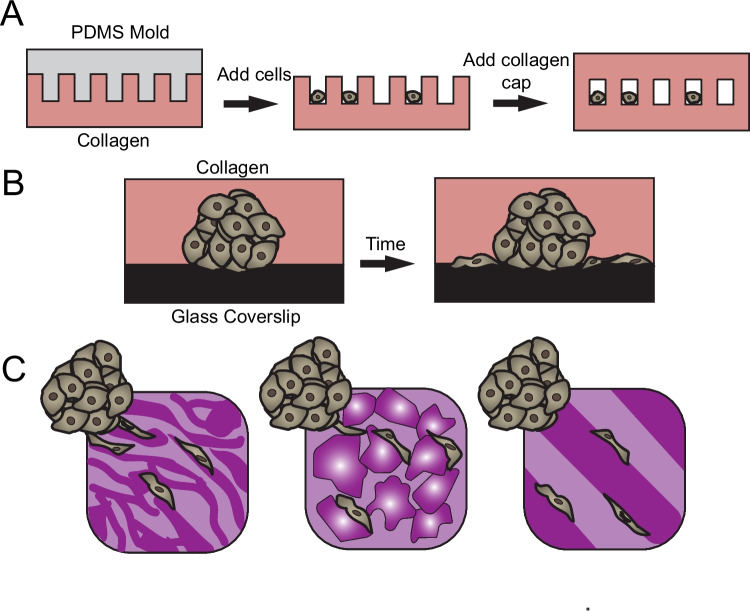
Strategies to introduce microtracks in vitro. Fabrication strategies to assess cell migration through microtracks include higher-order microchannel fabrication (**A**) or the engineering of interfaces between hydrogels and glass (**B**), bicontinuous hydrogels (**C**-left panel), particle composites (**C**-middle panel), and sandwich models (**C**-right panel)

Within the body, interfaces are found at the boundary of tissues, such as with myelinated axon tracks and perivascular spaces within the grey cortex of the brain, mammary gland ducts, and the collagen bundles between adipose tissues and within the developing tendon [136, 137]. Cancer cell metastasis may occur along preformed tracks with minimal resistance, with cells adapting their shapes to these preformed tracks [138]. Similarly, meniscal fibrochondrocytes rely on matrix microstructure to guide infiltration into the tissue, with cells found within gaps between dense collagen bundles of the meniscus [139]. The exploration of these preformed tracks in vitro has been limited to microtracks in collagen created through laser ablation/microfabrication or exploiting the interfaces between hydrogels and glass coverslips [140, 141] (Fig. 5A, B).

As mentioned, the use of collagen substrates limits the ability to decouple various properties of the extracellular environment. Until now, understanding cell migration with these interfaces was limited by the lack of fully defined systems where microtracks are included. We recently introduced an innovative top-down approach to engineer microinterfaces into hydrogels by using bicontinuous hydrogels, structures that consist of two interconnected, but discrete domains (Fig. 3D) [86]. When migrating, cells were found along the interfaces between these domains and found to migrate at higher speeds and distances compared to hydrogels with uniform structures. In this work, we also explored how the modularity of this mechanism can be used across a wide range of hydrogels for cell migration (Fig. 5C, right panel). Other ways to explore migration along microinterfaces include the bottom-up assembly of particles within composite hydrogels, whereby the interfaces between the particles and the continuous phase lend themselves to migration (Fig. 5C, middle panel). For full control over composition and mechanics of each layer, layered hydrogels can be progressively fabricated to allow cells to infiltrate along interfaces between layers (Fig. 5C, right panel). These strategies enable cell migration through the recapitulation and harnessing of microinterfaces in engineered environments.

## Conclusions

Cell migration is critical across many biological environments, from tissue development, health, and disease, where an interplay between cell machinery and extracellular environments drives migratory behavior. Hydrogels are useful as in vitro platforms to explore what features of the extracellular space may support cell migration and have highlighted the importance of numerous cues, including degradation, porosity, and viscoelasticity. Preformed tracks are another cue that supports cell migration in vivo, but only recently have engineering approaches begun to explore and harness this to control migration. These findings have supported further development of novel approaches to introduce these cues into hydrogels to guide tissue repair and regeneration in vivo, towards the translation of hydrogels in medicine. There is great promise for further development in this field, both in the understanding of signals that guide and support migration and the translation of these features into new therapies.

## Data Availability

There is no original research data within this review article.

## References

[1] Franz CM, Jones GE, Ridley AJ. Cell migration in development and disease. Dev Cell. 2002;2(2):153–8. 11832241 10.1016/s1534-5807(02)00120-x

[2] Chaudhuri O, et al. Effects of extracellular matrix viscoelasticity on cellular behaviour. Nature. 2020;584(7822):535–46. 32848221 10.1038/s41586-020-2612-2 PMC7676152

[3] Pijuan J et al. In vitro cell migration, invasion, and adhesion assays: from cell imaging to data analysis. Front Cell Dev Biol 2019;7:107. 10.3389/fcell.2019.00107 PMC6587234 31259172

[4] Oner A, Kobold S. Transwell migration assay to interrogate human CAR-T cell chemotaxis. STAR Protoc. 2022;3(4):101708. 36136753 10.1016/j.xpro.2022.101708 PMC9508471

[5] Fanfone D, et al. Confined migration promotes cancer metastasis through resistance to anoikis and increased invasiveness. eLife. 2022;11:e73150. 35256052 10.7554/eLife.73150 PMC8903834

[6] Cai G, et al. IGFBP1 sustains cell survival during spatially-confined migration and promotes tumor metastasis. Adv Sci. 2023;10(21):2206540. 10.1002/advs.202206540 PMC10375137 37296072

[7] Xu KL, Mauck RL, Burdick JA. Modeling development using hydrogels. Development. 2023;150(13):dev201527. 10.1242/dev.201527 PMC10323241 37387575

[8] Rehmann MS, et al. Tuning and predicting mesh size and protein release from step growth hydrogels. Biomacromol. 2017;18(10):3131–42. 10.1021/acs.biomac.7b00781 PMC6699171 28850788

[9] Alvarez-González B, et al. Cytoskeletal mechanics regulating amoeboid cell locomotion. Appl Mech Rev. 2014;66(5):0508041–05080414. 25328163 10.1115/1.4026249 PMC4201387

[10] Lämmermann T, Sixt M. Mechanical modes of ‘amoeboid’ cell migration. Curr Opin Cell Biol. 2009;21(5):636–44. 19523798 10.1016/j.ceb.2009.05.003

[11] Lämmermann T, et al. Rapid leukocyte migration by integrin-independent flowing and squeezing. Nature. 2008;453(7191):51–5. 18451854 10.1038/nature06887

[12] Petrie RJ, Koo H, Yamada KM. Generation of compartmentalized pressure by a nuclear piston governs cell motility in a 3D matrix. Science. 2014;345(6200):1062–5. 25170155 10.1126/science.1256965 PMC5248932

[13] Yamada KM, Sixt M. Mechanisms of 3D cell migration. Nat Rev Mol Cell Biol. 2019;20(12):738–52. 31582855 10.1038/s41580-019-0172-9

[14] Panková K, et al. The molecular mechanisms of transition between mesenchymal and amoeboid invasiveness in tumor cells. Cell Mol Life Sci. 2010;67(1):63–71. 19707854 10.1007/s00018-009-0132-1 PMC2801846

[15] SenGupta S, Parent CA, Bear JE. The principles of directed cell migration. Nat Rev Mol Cell Biol. 2021;22(8):529–47. 33990789 10.1038/s41580-021-00366-6 PMC8663916

[16] Kriebel PW, et al. Extracellular vesicles direct migration by synthesizing and releasing chemotactic signals. J Cell Biol. 2018;217(8):2891–910. 29884750 10.1083/jcb.201710170 PMC6080930

[17] Dowdell A, et al. Competition between chemoattractants causes unexpected complexity and can explain negative chemotaxis. Curr Biol. 2023;33(9):1704-1715.e3. 37001521 10.1016/j.cub.2023.03.006

[18] Qu F, et al. Programmed biomolecule delivery to enable and direct cell migration for connective tissue repair. Nat Commun. 2017;8(1):1780. 29176654 10.1038/s41467-017-01955-w PMC5701126

[19] Del Amo C, et al. Quantifying 3D chemotaxis in microfluidic-based chips with step gradients of collagen hydrogel concentrations. Integr Biol. 2017;9(4):339–49. 10.1039/c7ib00022g 28300261

[20] Yoon D, et al. Study on chemotaxis and chemokinesis of bone marrow-derived mesenchymal stem cells in hydrogel-based 3D microfluidic devices. Biomaterials Research. 2016;20(1):25. 27489724 10.1186/s40824-016-0070-6 PMC4971648

[21] Kasapidou PM, et al. Hyaluronic acid-based hydrogels loaded with chemoattractant and anticancer drug – new formulation for attracting and tackling glioma cells. Soft Matter. 2021;17(48):10846–61. 34806746 10.1039/d1sm01003d

[22] He R-Y, et al. Ovarian cancer cell adhesion/migration dynamics on micro-structured laminin gradients fabricated by multiphoton excited photochemistry. Bioengineering. 2015;2(3):139–59. 28952475 10.3390/bioengineering2030139 PMC5597181

[23] Wen JH, et al. Haptotaxis is cell type specific and limited by substrate adhesiveness. Cell Mol Bioeng. 2015;8(4):530–42. 26640598 10.1007/s12195-015-0398-3 PMC4667988

[24] Schwarz J, et al. Dendritic cells interpret haptotactic chemokine gradients in a manner governed by signal-to-noise ratio and dependent on GRK6. Curr Biol. 2017;27(9):1314–25. 28457871 10.1016/j.cub.2017.04.004

[25] Ren G, Liu B. Global classical solvability in a three-dimensional haptotaxis system modeling oncolytic virotherapy. Mathematical Methods in the Applied Sciences. 2021;44(11):9275–91.

[26] Hadjipanayi E, Mudera V, Brown RA. Guiding cell migration in 3D: A collagen matrix with graded directional stiffness. Cell Motil. 2009;66(3):121–8. 10.1002/cm.20331 19170223

[27] Zaman MH, et al. Migration of tumor cells in 3D matrices is governed by matrix stiffness along with cell-matrix adhesion and proteolysis. Proc Natl Acad Sci. 2006;103(29):10889–94. 16832052 10.1073/pnas.0604460103 PMC1544144

[28] Wang M, et al. Effect of three-dimensional ECM stiffness on cancer cell migration through regulating cell volume homeostasis. Biochem Biophys Res Commun. 2020;528(3):459–65. 32505356 10.1016/j.bbrc.2020.05.182

[29] Chang J, et al. Increased stiffness inhibits invadopodia formation and cell migration in 3D. Biophys J. 2020;119(4):726–36. 32697977 10.1016/j.bpj.2020.07.003 PMC7451915

[30] Iwasa SN, Popovic MR, Morshead CM. Skin-derived precursor cells undergo substrate-dependent galvanotaxis that can be modified by neighbouring cells. Stem Cell Research. 2018;31:95–101. 30059907 10.1016/j.scr.2018.07.019

[31] Zhu K, et al. Electric fields at breast cancer and cancer cell collective galvanotaxis. Sci Rep. 2020;10(1):8712. 32457381 10.1038/s41598-020-65566-0 PMC7250931

[32] Nakajima K-i et al. KCNJ15/Kir4.2 couples with polyamines to sense weak extracellular electric fields in galvanotaxis. Nature Commun. 2015;6(1): 8532. 10.1038/ncomms9532 PMC4603535 26449415

[33] Song B, et al. Application of direct current electric fields to cells and tissues in vitro and modulation of wound electric field in vivo. Nat Protoc. 2007;2(6):1479–89. 17545984 10.1038/nprot.2007.205

[34] Huang Y-J, et al. Cellular microenvironment modulates the galvanotaxis of brain tumor initiating cells. Sci Rep. 2016;6(1):21583. 26898606 10.1038/srep21583 PMC4761929

[35] Hu Q, et al. A flexible rapid self-assembly scaffold-net DNA hydrogel exhibiting cell mobility control. Mater Today Chem. 2022;23:100680.

[36] Cao W, et al. Migration of endothelial cells into photo-responsive hydrogels with tunable modulus under the presence of pro-inflammatory macrophages. Regenerative Biomater. 2019;6(5):259–67. 10.1093/rb/rbz025 PMC6783701 31616563

[37] Ravanbakhsh H, Bao G, Mongeau L. Carbon nanotubes promote cell migration in hydrogels. Sci Rep. 2020;10(1):2543. 32054957 10.1038/s41598-020-59463-9 PMC7018775

[38] Chen Y, et al. Tailorable hydrogel improves retention and cardioprotection of intramyocardial transplanted mesenchymal stem cells for the treatment of acute myocardial infarction in mice. J Am Heart Assoc. 2020;9(2):e013784. 31955638 10.1161/JAHA.119.013784 PMC7033822

[39] Jiang G, et al. A 3D-printed PRP-GelMA hydrogel promotes osteochondral regeneration through M2 macrophage polarization in a rabbit model. Acta Biomater. 2021;128:150–62. 33894346 10.1016/j.actbio.2021.04.010

[40] Ducker M, et al. A semi-automated and scalable 3D spheroid assay to study neuroblast migration. Stem Cell Rep. 2020;15(3):789–802. 10.1016/j.stemcr.2020.07.012 PMC7486343 32763162

[41] Chahal AS, et al. Human platelet lysate-loaded poly(ethylene glycol) hydrogels induce stem cell chemotaxis in vitro. Biomacromol. 2021;22(8):3486–96. 10.1021/acs.biomac.1c00573 PMC8382254 34314152

[42] Gao G, et al. Construction of a novel in vitro atherosclerotic model from geometry-tunable artery equivalents engineered via in-bath coaxial cell printing. Adv Func Mater. 2021;31(10):2008878.

[43] Ingram PN, et al. An accessible organotypic microvessel model using iPSC-derived endothelium. Adv Healthcare Mater. 2018;7(2):1700497. 10.1002/adhm.201700497 PMC6402823 29364596

[44] Shabalina EY, et al. The matrix-dependent 3D spheroid model of the migration of non-small cell lung cancer: a step towards a rapid automated screening. Front Mol Biosci. 2021;8:610407. 34422897 10.3389/fmolb.2021.610407 PMC8378843

[45] Tomasova L, et al. Advanced 2D/3D cell migration assay for faster evaluation of chemotaxis of slow-moving cells. PLoS ONE. 2019;14(7):e0219708. 31314801 10.1371/journal.pone.0219708 PMC6636736

[46] Qazi TH, et al. Anisotropic rod-shaped particles influence injectable granular hydrogel properties and cell invasion. Adv Mater. 2022;34(12):2109194. 10.1002/adma.202109194 PMC8957565 34932833

[47] McArdle TJ, Ogle BM, Noubissi FK. Moving upwards: a simple and flexible in vitro three-dimensional invasion assay protocol. J Vis Exp, 2018;(133):e56568. 10.3791/56568 PMC5931696 29578529

[48] Song KH, et al. Influence of fiber stiffness on meniscal cell migration into dense fibrous networks. Adv Healthc Mater. 2020;9(8):e1901228. 31867881 10.1002/adhm.201901228 PMC7274873

[49] Brekhman V, Neufeld G. A novel asymmetric 3D in-vitro assay for the study of tumor cell invasion. BMC Cancer. 2009;9(1):415. 19948022 10.1186/1471-2407-9-415 PMC2791776

[50] Visweshwaran SP, Gautreau A. Analysis of random migration of cancer cells in 3D. Bio Protoc. 2020;10(1):e3482. 33654715 10.21769/BioProtoc.3482 PMC7842704

[51] Visweshwaran SP, Maritzen T. A simple 3D cellular chemotaxis assay and analysis workflow suitable for a wide range of migrating cells. MethodsX. 2019;6:2807–21. 31871915 10.1016/j.mex.2019.11.001 PMC6909357

[52] Miura K. Tracking movement in cell biology. Adv Biochem Eng Biotechnology. 2005;95:267–95. 10.1007/b102218 16080272

[53] Lee SY, et al. High throughput 3D cell migration assay using micropillar/microwell chips. Molecules. 2022;27(16):5306. 36014542 10.3390/molecules27165306 PMC9416089

[54] Timm DM, et al. A high-throughput three-dimensional cell migration assay for toxicity screening with mobile device-based macroscopic image analysis. Sci Rep. 2013;3(1):3000. 24141454 10.1038/srep03000 PMC3801146

[55] Sayer S, et al. Guiding cell migration in 3D with high-resolution photografting. Sci Rep. 2022;12(1):8626. 35606455 10.1038/s41598-022-11612-y PMC9126875

[56] Gjorevski N, et al. Tissue geometry drives deterministic organoid patterning. Science. 2022;375(6576):eaaw9021. 34990240 10.1126/science.aaw9021 PMC9131435

[57] Kuo C-Y, et al. Development of a 3D printed, bioengineered placenta model to evaluate the role of trophoblast migration in preeclampsia. ACS Biomater Sci Eng. 2016;2(10):1817–26. 33440479 10.1021/acsbiomaterials.6b00031

[58] Motealleh A, Schulten A, Kehr NS. 3D printed step-gradient composite hydrogels for directed migration and osteogenic differentiation of human bone marrow-derived mesenchymal stem cells. Nano Select. 2022;3(2):411–24.

[59] Piard C, et al. 3D printed HUVECs/MSCs cocultures impact cellular interactions and angiogenesis depending on cell-cell distance. Biomaterials. 2019;222:119423. 31442885 10.1016/j.biomaterials.2019.119423 PMC6745276

[60] Abhyankar VV, et al. A platform for assessing chemotactic migration within a spatiotemporally defined 3D microenvironment. Lab Chip. 2008;8(9):1507–15. 18818806 10.1039/b803533d PMC2804469

[61] Nishimura KY, Isseroff RR, Nuccitelli R. Human keratinocytes migrate to the negative pole in direct current electric fields comparable to those measured in mammalian wounds. J Cell Sci. 1996;109(1):199–207. 8834804 10.1242/jcs.109.1.199

[62] Li BB, et al. Cell invasion in digital microfluidic microgel systems. Sci Adv. 2020;6(29):eaba9589. 32832633 10.1126/sciadv.aba9589 PMC7439438

[63] Lugo-Cintrón KM, et al. Breast fibroblasts and ECM components modulate breast cancer cell migration through the secretion of mmps in a 3D microfluidic co-culture model. Cancers. 2020;12(5):1173. 10.3390/cancers12051173. 32384738 10.3390/cancers12051173 PMC7281408

[64] Saha B, et al. Human tumor microenvironment chip evaluates the consequences of platelet extravasation and combinatorial antitumor-antiplatelet therapy in ovarian cancer. Sci Adv. 2021;7(30):eabg5283. 34290095 10.1126/sciadv.abg5283 PMC8294767

[65] Kramer N, et al. In vitro cell migration and invasion assays. Mutation Res/Rev Mutation Res. 2013;752(1):10–24. 10.1016/j.mrrev.2012.08.001 22940039

[66] Solbu AA, et al. Assessing cell migration in hydrogels: an overview of relevant materials and methods. Mater Today Bio. 2023;18:100537. 36659998 10.1016/j.mtbio.2022.100537 PMC9842866

[67] Petrie RJ, Yamada KM. At the leading edge of three-dimensional cell migration. J Cell Sci. 2012;125(Pt 24):5917–26. 23378019 10.1242/jcs.093732 PMC4067260

[68] Yue B. Biology of the extracellular matrix: an overview. J Glaucoma. 2014;23(8 Suppl 1):S20–3. 25275899 10.1097/IJG.0000000000000108 PMC4185430

[69] Lu P, et al. Extracellular matrix degradation and remodeling in development and disease. Cold Spring Harb Perspect Biol. 2011;3(12):a005058. 21917992 10.1101/cshperspect.a005058 PMC3225943

[70] van Hinsbergh VWM, Koolwijk P. Endothelial sprouting and angiogenesis: matrix metalloproteinases in the lead. Cardiovasc Res. 2008;78(2):203–12. 18079100 10.1093/cvr/cvm102

[71] Murphy G, Gavrilovic J. Proteolysis and cell migration: creating a path? Curr Opin Cell Biol. 1999;11(5):614–21. 10508651 10.1016/s0955-0674(99)00022-8

[72] Wiesner C, et al. KIF5B and KIF3A/KIF3B kinesins drive MT1-MMP surface exposure, CD44 shedding, and extracellular matrix degradation in primary macrophages. Blood. 2010;116(9):1559–69. 20505159 10.1182/blood-2009-12-257089

[73] Bayarmagnai B, et al. Invadopodia-mediated ECM degradation is enhanced in the G1 phase of the cell cycle. J Cell Sci, 2019;132(20):jcs227116. 10.1242/jcs.227116 PMC6826011 31533971

[74] Gobin AS, West JL. Cell migration through defined, synthetic extracellular matrix analogues. FASEB J. 2002;16(7):751–3. 11923220 10.1096/fj.01-0759fje

[75] West JL, Hubbell JA. Polymeric biomaterials with degradation sites for proteases involved in cell migration. Macromolecules. 1999;32(1):241–4.

[76] Ehrbar M, et al. Elucidating the role of matrix stiffness in 3D cell migration and remodeling. Biophys J. 2011;100(2):284–93. 21244824 10.1016/j.bpj.2010.11.082 PMC3021668

[77] Trappmann B, et al. Matrix degradability controls multicellularity of 3D cell migration. Nat Commun. 2017;8(1):371. 28851858 10.1038/s41467-017-00418-6 PMC5575316

[78] Schultz KM, Kyburz KA, Anseth KS. Measuring dynamic cell–material interactions and remodeling during 3D human mesenchymal stem cell migration in hydrogels. Proc Natl Acad Sci. 2015;112(29):E3757–64. 26150508 10.1073/pnas.1511304112 PMC4517280

[79] Legant WR, et al. Measurement of mechanical tractions exerted by cells in three-dimensional matrices. Nat Methods. 2010;7(12):969–71. 21076420 10.1038/nmeth.1531 PMC3056435

[80] Wade RJ, et al. Protease-degradable electrospun fibrous hydrogels. Nature. Communications. 2015;6(1):6639. 10.1038/ncomms7639 PMC4372144 25799370

[81] Li Y, Hoffman MD, Benoit DSW. Matrix metalloproteinase (MMP)-degradable tissue engineered periosteum coordinates allograft healing via early stage recruitment and support of host neurovasculature. Biomaterials. 2021;268:120535. 33271450 10.1016/j.biomaterials.2020.120535 PMC8110201

[82] Reing JE, et al. Degradation products of extracellular matrix affect cell migration and proliferation. Tissue Eng Part A. 2008;15(3):605–14. 10.1089/ten.tea.2007.0425 18652541

[83] Platel A, et al. Influence of the surface charge of PLGA nanoparticles on their in vitro genotoxicity, cytotoxicity, ROS production and endocytosis. J Appl Toxicol. 2016;36(3):434–44. 26487569 10.1002/jat.3247

[84] Wisdom KM, et al. Matrix mechanical plasticity regulates cancer cell migration through confining microenvironments. Nat Commun. 2018;9(1):4144. 30297715 10.1038/s41467-018-06641-z PMC6175826

[85] Seymour AJ, Shin S, Heilshorn SC. 3D printing of microgel scaffolds with tunable void fraction to promote cell infiltration. Adv Healthcare Mater. 2021;10(18):2100644. 10.1002/adhm.202100644 PMC8612872 34342179

[86] Xu KL, et al. Microinterfaces in biopolymer-based bicontinuous hydrogels guide rapid 3D cell migration. Nat Commun. 2024;15(1):2766. 38553465 10.1038/s41467-024-46774-y PMC10980809

[87] Wheatley BB, et al. An optimized transversely isotropic, hyper-poro-viscoelastic finite element model of the meniscus to evaluate mechanical degradation following traumatic loading. J Biomech. 2015;48(8):1454–60. 25776872 10.1016/j.jbiomech.2015.02.028 PMC4442040

[88] Fan W, et al. Matrix viscoelasticity promotes liver cancer progression in the pre-cirrhotic liver. Nature. 2024;626(7999):635–42. 38297127 10.1038/s41586-023-06991-9 PMC10866704

[89] Cameron AR, Frith JE, Cooper-White JJ. The influence of substrate creep on mesenchymal stem cell behaviour and phenotype. Biomaterials. 2011;32(26):5979–93. 21621838 10.1016/j.biomaterials.2011.04.003

[90] Charrier EE, et al. Control of cell morphology and differentiation by substrates with independently tunable elasticity and viscous dissipation. Nat Commun. 2018;9(1):449. 29386514 10.1038/s41467-018-02906-9 PMC5792430

[91] Chaudhuri O, et al. Substrate stress relaxation regulates cell spreading. Nat Commun. 2015;6(1):6365. 10.1038/ncomms7365 PMC4518451 25695512

[92] Gong Z, et al. Matching material and cellular timescales maximizes cell spreading on viscoelastic substrates. Proc Natl Acad Sci. 2018;115(12):E2686–95. 29507238 10.1073/pnas.1716620115 PMC5866566

[93] Lou J, et al. Stress relaxing hyaluronic acid-collagen hydrogels promote cell spreading, fiber remodeling, and focal adhesion formation in 3D cell culture. Biomaterials. 2018;154:213–22. 29132046 10.1016/j.biomaterials.2017.11.004

[94] Nam S, et al. Varying PEG density to control stress relaxation in alginate-PEG hydrogels for 3D cell culture studies. Biomaterials. 2019;200:15–24. 30743050 10.1016/j.biomaterials.2019.02.004 PMC6463514

[95] Yang B, et al. Enhanced mechanosensing of cells in synthetic 3D matrix with controlled biophysical dynamics. Nat Commun. 2021;12(1):3514. 34112772 10.1038/s41467-021-23120-0 PMC8192531

[96] Tang S, et al. Adaptable fast relaxing boronate-based hydrogels for probing cell–matrix interactions. Adv Sci. 2018;5(9):1800638. 10.1002/advs.201800638 PMC6145256 30250802

[97] Wu DT, et al. Hydrogel viscoelasticity modulates migration and fusion of mesenchymal stem cell spheroids. Bioeng Transl Med. 2023;8(3):e10464. 37206235 10.1002/btm2.10464 PMC10189430

[98] Elosegui-Artola A, et al. Matrix viscoelasticity controls spatiotemporal tissue organization. Nat Mater. 2023;22(1):117–27. 36456871 10.1038/s41563-022-01400-4 PMC10332325

[99] Nam S, et al. Strain-enhanced stress relaxation impacts nonlinear elasticity in collagen gels. Proc Natl Acad Sci. 2016;113(20):5492–7. 27140623 10.1073/pnas.1523906113 PMC4878492

[100] Wei Z, et al. Hydrogel network dynamics regulate vascular morphogenesis. Cell Stem Cell. 2020;27(5):798-812.e6. 32931729 10.1016/j.stem.2020.08.005 PMC7655724

[101] Carberry BJ, Rao VV, Anseth KS. Phototunable viscoelasticity in hydrogels through thioester exchange. Ann Biomed Eng. 2020;48(7):2053–63. 32020346 10.1007/s10439-020-02460-w PMC7334082

[102] Richardson BM, et al. Mechanobiological interactions between dynamic compressive loading and viscoelasticity on chondrocytes in hydrazone covalent adaptable networks for cartilage tissue engineering. Adv Healthcare Mater. 2021;10(9):2002030. 10.1002/adhm.202002030 PMC8785214 33738966

[103] Wolf K, et al. Collagen-based cell migration models in vitro and in vivo. Semin Cell Dev Biol. 2009;20(8):931–41. 19682592 10.1016/j.semcdb.2009.08.005 PMC4021709

[104] Denais CM, et al. Nuclear envelope rupture and repair during cancer cell migration. Science. 2016;352(6283):353–8. 27013428 10.1126/science.aad7297 PMC4833568

[105] Friedl P, Wolf K, Lammerding J. Nuclear mechanics during cell migration. Curr Opin Cell Biol. 2011;23(1):55–64. 21109415 10.1016/j.ceb.2010.10.015 PMC3073574

[106] Heo SJ, Song KH, Thakur S, Miller LM, Cao X, Peredo AP, Seiber BN, Qu F, Driscoll TP, Shenoy VB, Lakadamyali M. Nuclear softening expedites interstitial cell migration in fibrous networks and dense connective tissues. Science advances. 2020;6(25):eaax5083. 32596438 10.1126/sciadv.aax5083 PMC7304973

[107] Annabi N, et al. Controlling the porosity and microarchitecture of hydrogels for tissue engineering. Tissue Eng Part B Rev. 2010;16(4):371–83. 10.1089/ten.teb.2009.0639 PMC2946907 20121414

[108] Bodenberger N, et al. Evaluation of methods for pore generation and their influence on physio-chemical properties of a protein based hydrogel. Biotechnol Rep (Amst). 2016;12:6–12. 28352549 10.1016/j.btre.2016.09.001 PMC5361077

[109] Mooney DJ, et al. Novel approach to fabricate porous sponges of poly(d, l-lactic-co-glycolic acid) without the use of organic solvents. Biomaterials. 1996;17(14):1417–22. 8830969 10.1016/0142-9612(96)87284-x

[110] Liu YX, et al. Visualization of porosity and pore size gradients in electrospun scaffolds using laser metrology. PLoS ONE. 2023;18(3):e0282903. 36893193 10.1371/journal.pone.0282903 PMC9997878

[111] Stachewicz U, et al. Pore shape and size dependence on cell growth into electrospun fiber scaffolds for tissue engineering: 2D and 3D analyses using SEM and FIB-SEM tomography. Mater Sci Eng, C. 2019;95:397–408. 10.1016/j.msec.2017.08.076 30573264

[112] Lanno G-M, et al. Antibacterial porous electrospun fibers as skin scaffolds for wound healing applications. ACS Omega. 2020;5(46):30011–22. 33251437 10.1021/acsomega.0c04402 PMC7689890

[113] Baker BM, et al. Sacrificial nanofibrous composites provide instruction without impediment and enable functional tissue formation. Proc Natl Acad Sci. 2012;109(35):14176–81. 22872864 10.1073/pnas.1206962109 PMC3435214

[114] Nam J, et al. Improved cellular infiltration in electrospun fiber via engineered porosity. Tissue Eng. 2007;13(9):2249–57. 17536926 10.1089/ten.2006.0306 PMC4948987

[115] Ameer JM, Pr AK, Kasoju N. Strategies to tune electrospun scaffold porosity for effective cell response in tissue engineering. J Funct Biomater. 2019;10(3):30. 31324062 10.3390/jfb10030030 PMC6787600

[116] Wu J, Hong Y. Enhancing cell infiltration of electrospun fibrous scaffolds in tissue regeneration. Bioactive Mater. 2016;1(1):56–64. 10.1016/j.bioactmat.2016.07.001 PMC5883964 29744395

[117] Nih LR, et al. Injection of microporous annealing particle (MAP) hydrogels in the stroke cavity reduces gliosis and inflammation and promotes NPC migration to the lesion. Adv Mater. 2017;29(32):1606471. 10.1002/adma.201606471 PMC5595584 28650574

[118] Suturin AC, et al. Annealing high aspect ratio microgels into macroporous 3D scaffolds allows for higher porosities and effective cell migration. Adv Healthcare Mater. 2022;11(24):2200989. 10.1002/adhm.202200989 PMC11469137 36100464

[119] Cunha AF, et al. Cell response in free-packed granular systems. ACS Appl Mater Interfaces. 2022;14(36):40469–80. 36044384 10.1021/acsami.1c24095 PMC9773234

[120] Muir VG, et al. Influence of microgel fabrication technique on granular hydrogel properties. ACS Biomater Sci Eng. 2021;7(9):4269–81. 33591726 10.1021/acsbiomaterials.0c01612 PMC8966052

[121] Anderson AR, Nicklow E, Segura T. Particle fraction is a bioactive cue in granular scaffolds. Acta Biomater. 2022;150:111–27. 35917913 10.1016/j.actbio.2022.07.051 PMC10329855

[122] Mealy JE, et al. Injectable granular hydrogels with multifunctional properties for biomedical applications. Adv Mater. 2018;30(20):1705912. 10.1002/adma.201705912 29602270

[123] Wolf K, et al. Physical limits of cell migration: control by ECM space and nuclear deformation and tuning by proteolysis and traction force. J Cell Biol. 2013;201(7):1069–84. 23798731 10.1083/jcb.201210152 PMC3691458

[124] Wu P-H, et al. Three-dimensional cell migration does not follow a random walk. Proc Natl Acad Sci. 2014;111(11):3949–54. 24594603 10.1073/pnas.1318967111 PMC3964056

[125] van Helvert S, Friedl P. Strain stiffening of fibrillar collagen during individual and collective cell migration identified by AFM nanoindentation. ACS Appl Mater Interfaces. 2016;8(34):21946–55. 27128771 10.1021/acsami.6b01755

[126] Sadjadi Z, et al. Migration of Cytotoxic T lymphocytes in 3D collagen matrices. Biophys J. 2020;119(11):2141–52. 33264597 10.1016/j.bpj.2020.10.020 PMC7732778

[127] Olofsson PE, et al. A collagen-based microwell migration assay to study NK-target cell interactions. Sci Rep. 2019;9(1):10672. 31337806 10.1038/s41598-019-46958-3 PMC6650390

[128] Hayn A, Fischer T, Mierke CT. Inhomogeneities in 3D collagen matrices impact matrix mechanics and cancer cell migration. Front Cell Dev Biol. 2020;8:593879. 33251219 10.3389/fcell.2020.593879 PMC7674772

[129] Nicolas-Boluda A, et al. Tumor stiffening reversion through collagen crosslinking inhibition improves T cell migration and anti-PD-1 treatment. eLife. 2021;10:e58688. 34106045 10.7554/eLife.58688 PMC8203293

[130] Pajic-Lijakovic I, Milivojevic M, Clark AG. Collective cell migration on collagen-I networks: the impact of matrix viscoelasticity. Front Cell Dev Biol. 2022;10:901026. 35859899 10.3389/fcell.2022.901026 PMC9289519

[131] Salam N, et al. Assessment of migration of human MSCs through fibrin hydrogels as a tool for formulation optimisation. Materials (Basel). 2018;11(9):1781. 30235852 10.3390/ma11091781 PMC6164849

[132] Henke CA, et al. CD44-related chondroitin sulfate proteoglycan, a cell surface receptor implicated with tumor cell invasion, mediates endothelial cell migration on fibrinogen and invasion into a fibrin matrix. J Clin Investig. 1996;97(11):2541–52. 8647947 10.1172/JCI118702 PMC507340

[133] Tarafder S, et al. Engineered healing of avascular meniscus tears by stem cell recruitment. Sci Rep. 2018;8(1):8150. 29802356 10.1038/s41598-018-26545-8 PMC5970239

[134] Ronfard V, Barrandon Y. Migration of keratinocytes through tunnels of digested fibrin. Proc Natl Acad Sci. 2001;98(8):4504–9. 11274362 10.1073/pnas.071631698 PMC31864

[135] Chandran PL, Barocas VH. Microstructural mechanics of collagen gels in confined compression: poroelasticity, viscoelasticity, and collapse. J Biomech Eng. 2004;126(2):152–66. 15179845 10.1115/1.1688774

[136] Starborg T, et al. Chapter 17 Electron microscopy of collagen fibril structure in vitro and in vivo including three-dimensional reconstruction. In: Methods in Cell Biology. Academic Press; 2008. p. 319–45. 10.1016/S0091-679X(08)00417-2 18617041

[137] Gritsenko GP, Ilina O, Friedl P. Interstitial guidance of cancer invasion. J Pathol. 2012;226(2):185–99. 22006671 10.1002/path.3031

[138] Weigelin B, Bakker G-J, Friedl P. Intravital third harmonic generation microscopy of collective melanoma cell invasion. IntraVital. 2012;1(1):32–43. 29607252 10.4161/intv.21223 PMC5858865

[139] Qu F, et al. Maturation state and matrix microstructure regulate interstitial cell migration in dense connective tissues. Sci Rep. 2018;8(1):3295. 29459687 10.1038/s41598-018-21212-4 PMC5818574

[140] Ilina O, et al. Cell–cell adhesion and 3D matrix confinement determine jamming transitions in breast cancer invasion. Nat Cell Biol. 2020;22(9):1103–15. 32839548 10.1038/s41556-020-0552-6 PMC7502685

[141] Kraning-Rush CM, et al. Microfabricated collagen tracks facilitate single cell metastatic invasion in 3D. Integr Biol. 2013;5(3):606–16. 10.1039/c3ib20196a PMC3601578 23388698

